# Supplementary material for: Probing the dynamic landscape of peptides in molecular assemblies by synergized NMR experiments and MD simulations
Source: Commun Chem. 2024 Feb 13;7:28. doi: 10.1038/s42004-024-01115-4 (PMC10864328; doi:10.1038/s42004-024-01115-4)
Supplement: Supplementary file 2 — Supplementary Information [file 42004_2024_1115_MOESM2_ESM.pdf]

SUPPLEMENTARY INFORMATION: Probing the dynamic landscape of peptides in molecular assemblies by synergized NMR experiments and MD simulations

Ricky Nencini[1,2], Morgan L. G. Regnier[1], Sofia M. Backlund[1], Efstathia Mantzari[1], Cory D. Dunn[1], and O. H. Samuli Ollila[1,3,*]

[1]Institute of Biotechnology, University of Helsinki, Helsinki, Finland
[2]Division of Pharmaceutical Biosciences, Faculty of Pharmacy, University of Helsinki, Helsinki, Finland
[3]VTT Technical Research Centre of Finland, Espoo, Finland
[*]samuli.ollila@helsinki.fi

Orientation of two peptides in a micelle with respect to each other

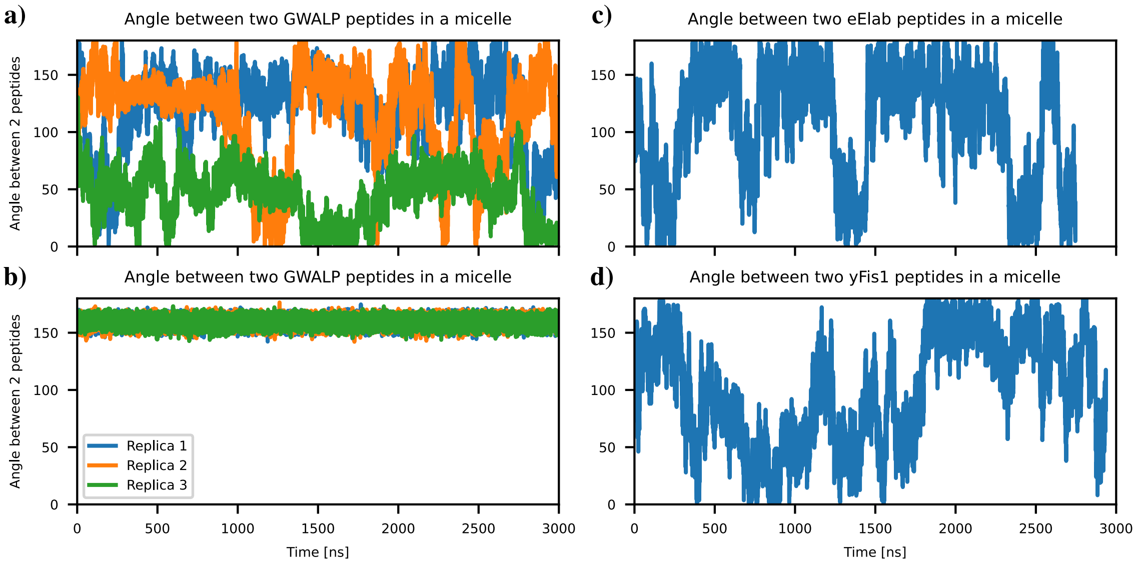

Supplementary Figure 1. Angle between the principal axes of the two peptides in a micelle for a) freely rotating GWALP peptides in a micelle with 70 SDS molecules, b) GWALP peptides rotating together in a micelle with 70 SDS molecules, c) eElab peptides in a micelle with 50 SDS molecules, and d) yFis1 peptides in a micelle with 50 SDS molecules.

Full dynamic landscape of peptides

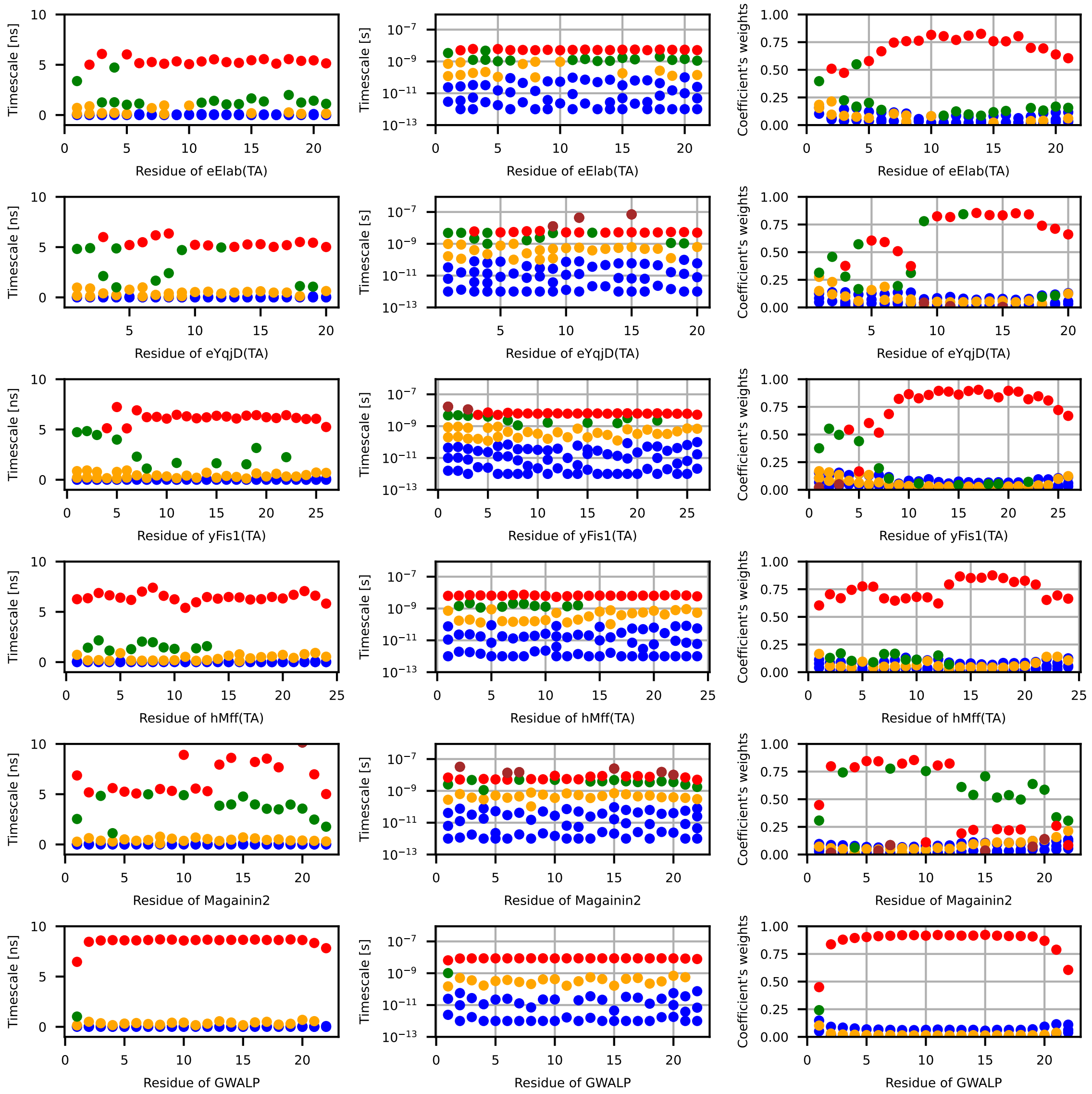

Supplementary Figure 2. Full dynamic landscape of studied peptides from the simulations in the best agreement with experiments in Fig. 4. Left: Timescales (τ_i) with non-zero weights between 0-10 ns shown using a linear scale for the y-axis. Middle: All observed timescales with non-zero weights are shown using a logarithmic scale for the y-axis. Right: Weights (α_i) for the timescales in the left and middle columns. Colour code: timescales smaller than 100 ps are shown in blue, timescales between 100 ps and 1 ns are in yellow, timescales between 1 ns and 5 ns are in green, timescales between 5 ns and 10 ns are red and timescales bigger than 10 ns (not shown in the plots on left) are shown in brown.

Overall rotation of SDS molecules in micelles

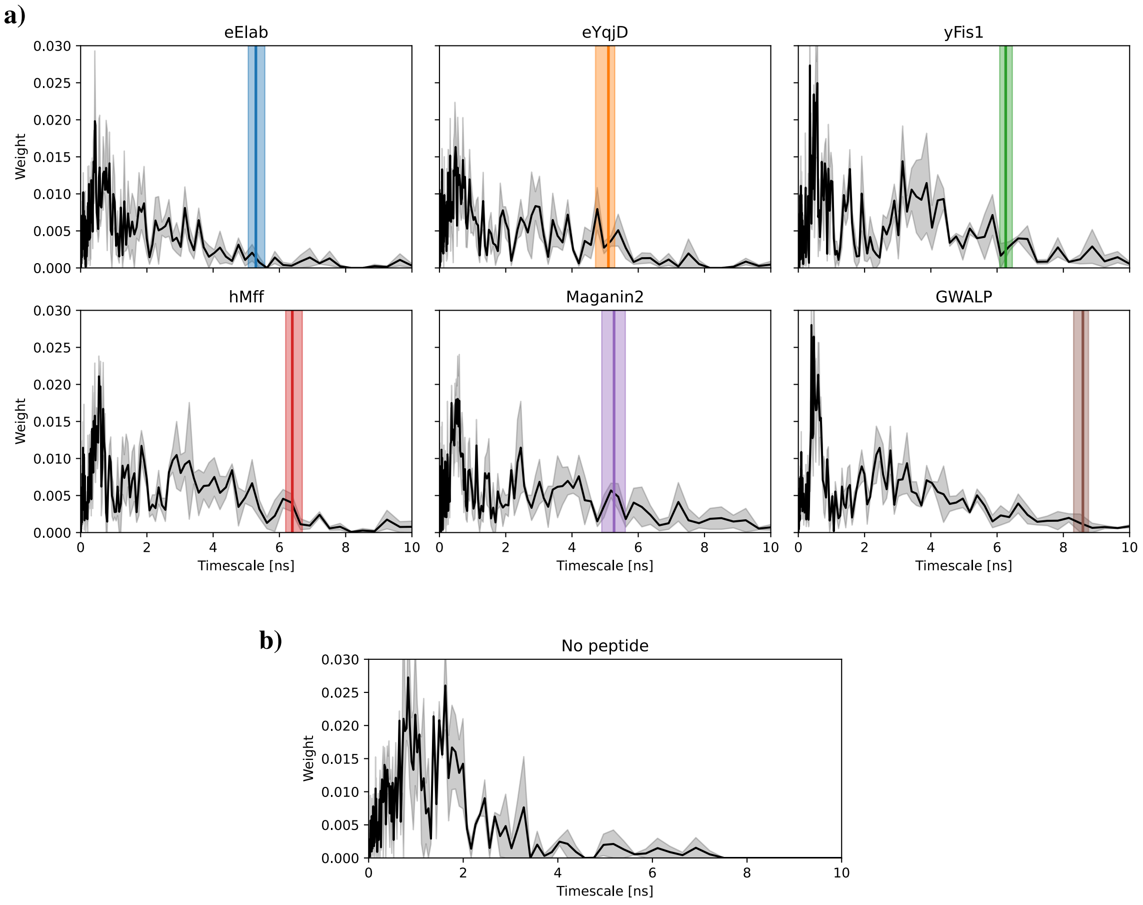

Supplementary Figure 3. Overall rotation timescales for SDS molecules estimated from the vector between the center of mass of micelle and sulfur atom. Distributions of timescales calculated from all SDS molecules in a) systems with peptides in micelles and b) SDS micelle without any peptides. Averages over 3 replicas are shown by the solid line and errors of the mean by the shaded regions. Vertical colored solid lines show the average dominant timescale of the given peptide calculated from residues with the dominant timescale having weight larger than 0.75. All the residues fulfilling these conditions lay within the shaded regions.

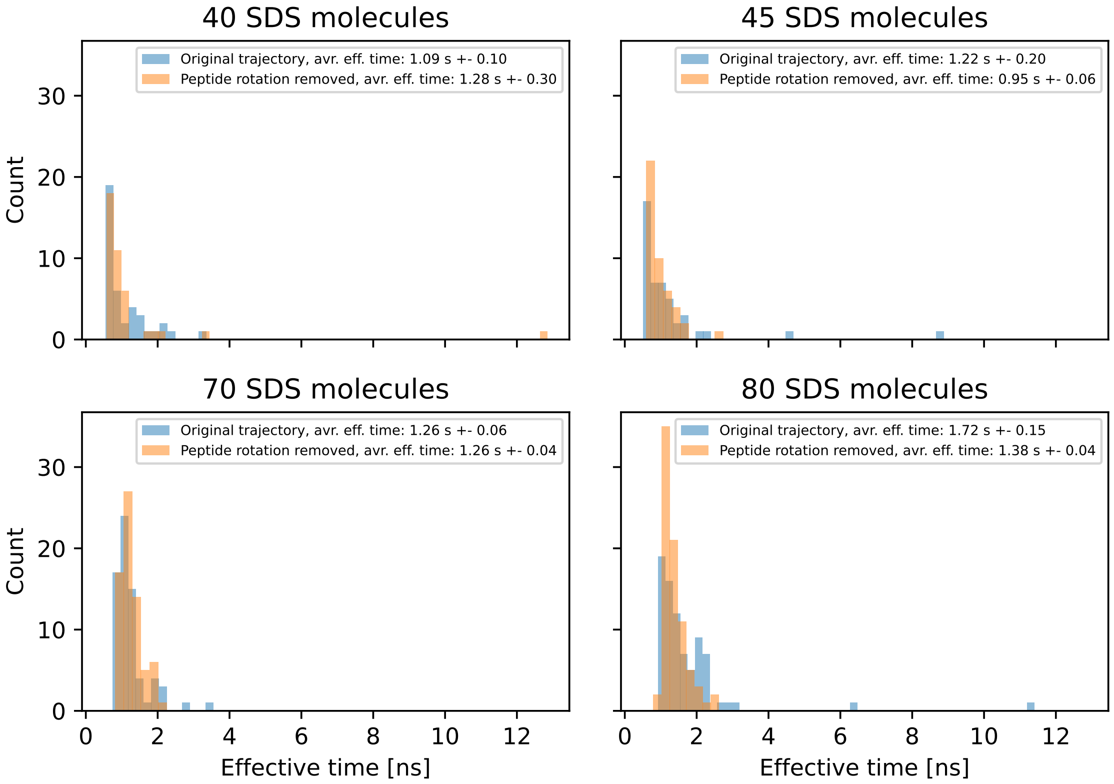

Supplementary Figure 4. Effective correlation times of overall SDS molecule rotations in micelles with monomeric GWALP peptides with different sizes estimated from the vector between the center of mass of micelle and sulfur atom. Distributions of timescales calculated from all SDS molecules in original trajectories are compared with the results from trajectories where the peptide rotation was removed.

Spin relaxation times as a function of micelle molecular weight

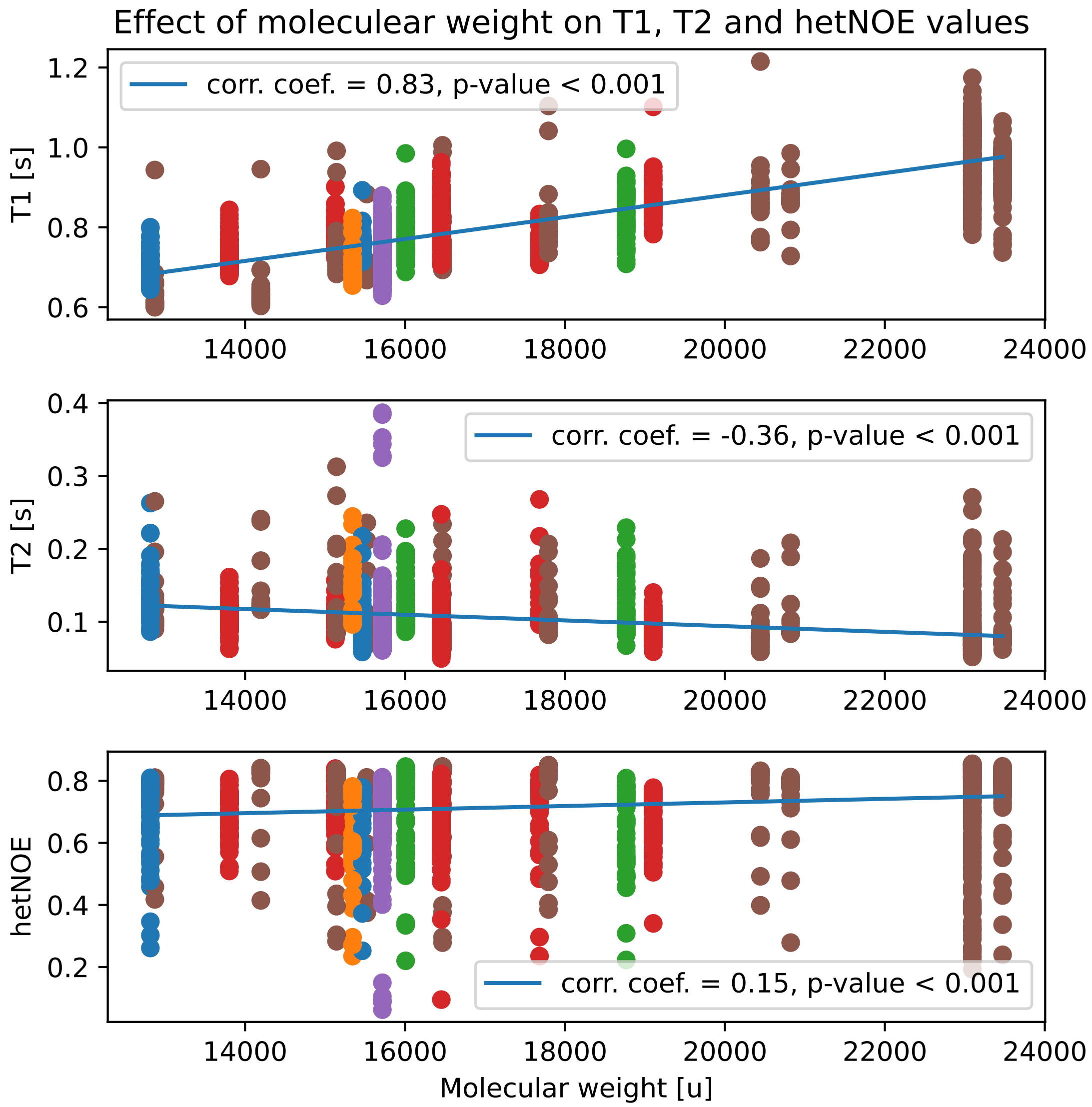

Supplementary Figure 5. Spin relaxation times as a function of total molecular weight calculated from all systems. The colors refer to different peptides as in figures in the main text.

Dependence of spin relaxation times on simulation box size

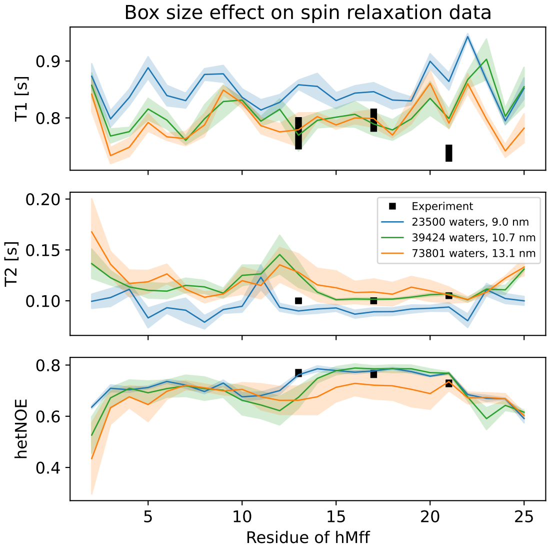

Supplementary Figure 6. Effect of MD simulation box size on spin relaxation times. Spin relaxation times calculated from hMff(TA) simulations with different simulation box sizes.

Determining the SDS molecules belonging to a micelle

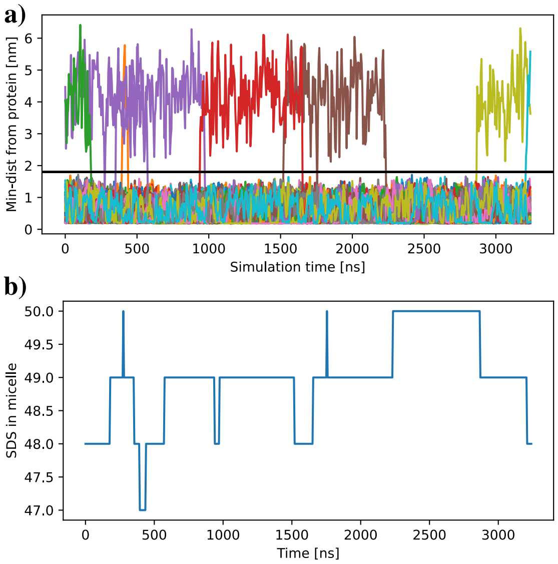

Supplementary Figure 7. Determination of SDS molecules within a micelle exemplified for eYqjD simulation with 50 SDS molecules (replica 1). a) Minimum distance between any SDS atom and any peptide atom as a function of time for each SDS molecule labelled with different colours. SDS molecules with a distance below the selected cut-off value were considered to be a part of a micelle. The selected cut-off for this system, 1.8 nm, is shown with the horizontal black line. b) The number of SDS molecules belonging to the micelle as a function of time.

Simulated systems

Supplementary Table 1. Simulated systems with a peptide in a SDS micelle using CHARMM36 force field with OPC water model at 310 K.

System	Length [ns]	SDS	Na^+	Water molecules	Micelle size [a)]	Starting configuration [b)]	Reference for the data[c]
eElaB(TA) 40 SDS replica1	4019	40	38	39442	39.0	CHARMM-GUI	17
eElaB(TA) 40 SDS replica2	3000	40	38	39442	38.6	eElaB(TA) 40 SDS replica1, 1500 ns	18
eElaB(TA) 40 SDS replica3	3000	40	38	39442	39.0	eElaB(TA) 40 SDS replica1, 3000 ns	19
eElaB(TA) 50 SDS replica2	3000	50	48	39442	48.4	eElaB(TA) 50 SDS replica1, 500 ns	20
eElaB(TA) 50 SDS replica3	3000	50	48	39442	48.6	eElaB(TA) 50 SDS replica1, 1000 ns	21
eYqjD(TA) 50 SDS replica1	3248	50	48	39446	49.0	CHARMM-GUI	22
eYqjD(TA) 50 SDS replica2	3000	50	48	39446	48.5	eYqjD(TA) 50 SDS replica1, 750 ns	23
eYqjD(TA) 50 SDS replica3	3000	50	48	39446	48.3	eYqjD(TA) 50 SDS replica1, 1500 ns	24
yFis1(TA) 50 SDS replica1	2885	50	45	39475	49.1	CHARMM-GUI	25
yFis1(TA) 50 SDS replica2	3000	50	45	39475	48.5	yFis1(TA) 50 SDS replica1, 750 ns	26
yFis1(TA) 50 SDS replica3	3000	50	45	39475	48.6	yFis1(TA) 50 SDS replica1, 1500 ns	27
hMff(TA) 40 SDS replica1	2966	40	37	39424	39.0	CHARMM-GUI	28
hMff(TA) 40 SDS replica2	3000	40	37	39424	39.4	hMff(TA) 40 SDS replica1, 750 ns	29
hMff(TA) 40 SDS replica3	3000	40	37	39424	38.5	hMff(TA) 40 SDS replica1, 1500 ns	30
hMff(TA) 45 SDS replica1	2940	45	42	39424	43.8	CHARMM-GUI	31
hMff(TA) 45 SDS replica2	3000	45	42	39424	43.7	hMff(TA) 45 SDS replica1, 750 ns	32
hMff(TA) 45 SDS replica3	3000	45	42	39424	43.9	hMff(TA) 45 SDS replica1, 1500 ns	33
hMff(TA) 50 SDS replica1	2948	50	47	39424	48.6	CHARMM-GUI	34
hMff(TA) 50 SDS replica2	3000	50	47	39424	48.2	hMff(TA) 50 SDS replica1, 750 ns	35
hMff(TA) 50 SDS replica3	3000	50	47	39424	47.7	hMff(TA) 50 SDS replica1, 1500 ns	36
hMff(TA) 50 SDS replica1 - small	3000	50	47	23500	49.4	hMff(TA) 50 SDS replica1, 1000 ns	37
hMff(TA) 50 SDS replica2 - small	3000	50	47	23500	49.2	hMff(TA) 50 SDS replica1 - small, 1000 ns	38
hMff(TA) 50 SDS replica3 - small	3000	50	47	23500	49.4	hMff(TA) 50 SDS replica1 - small, 2000 ns	39
hMff(TA) 50 SDS replica1 - big	3000	50	47	73801	46.5	hMff(TA) 50 SDS replica1, 2900 ns [e)]	40
hMff(TA) 50 SDS replica1 - big	3000	50	47	73801	45.5	hMff(TA) 50 SDS replica2, 3000 ns [e)]	41
hMff(TA) 50 SDS replica1 - big	3000	50	47	73801	46.0	hMff(TA) 50 SDS replica3, 23000 ns [e)]	42
hMff(TA) 60 SDS replica1	3153	60	57	25891	59.2	CHARMM-GUI	43
hMff(TA) 60 SDS replica2	3000	60	57	25891	59.4	hMff(TA) 60 SDS replica1, 750 ns	44
hMff(TA) 60 SDS replica3	3000	60	57	25891	58.6	hMff(TA) 60 SDS replica1, 1500 ns	45
Mageinin2 50 SDS replica1	2996	50	47	32836	36.3	CHARMM-GUI	46
Mageinin2 50 SDS replica2	3000	50	47	32836	38.5	Mageinin2 50 SDS replica1, 750 ns	47
Mageinin2 50 SDS replica3	3000	50	47	32836	43.9	Mageinin2 50 SDS replica1, 1500 ns	48
Mageinin2 50 SDS replica4	3000	50	47	32836	48.8	Mageinin2 50 SDS replica3, 2000 ns	49
Mageinin2 50 SDS replica5	3000	50	47	32836	48.4	Mageinin2 50 SDS replica3, 2500 ns	50
Mageinin2 50 SDS replica6	3000	50	47	32836	49.1	Mageinin2 50 SDS replica3, 3000 ns	51
GWALP 40 SDS replica1	1435	40	40	23868	39.6	CHARMM-GUI	52
GWALP 45 SDS replica1	1435	45	45	23868	44.7	CHARMM-GUI	53
GWALP 50 SDS replica1	1411	50	50	23868	49.3	CHARMM-GUI	54
GWALP 70 SDS replica1	3000	70	70	23523	69.1	GWALP dimer 70 SDS replica1, 1000 ns	55
GWALP 80 SDS replica1	3000	80	80	23868	79.5	Described in the note [d)]	56
GWALP 80 SDS replica2	3000	80	80	23868	78.7	CHARMM-GUI	57
GWALP 80 SDS replica3	3000	80	80	23868	79.1	GWALP 80 SDS replica2, 3000 ns	58

[a)] Average number of SDS molecules forming the micelle during simulation.
[b)] Origin of the starting configuration (name of the simulation, time of the frame).
[c)] All the simulation files including trajectories available from this reference.
[d)] 10 SDS molecules added to GWALP 70 SDS, equilibrated for 1 μs.
[e)] Box dimensions were increased and additional water was added using gmx solvate

Supplementary Table 2. Simulated systems with two peptides in an SDS micelle using CHARMM36 force field with the OPC water model at 310 K.

System	Length [ns]	SDS	Na^+	Water molecules	Micelle size [a)]	Starting configuration [b)]	Reference for the data[c]
eElaB(TA) 50 SDS replica1	2745	50	46	42994	49.1	Described in the text	59
yFis1(TA) 50 SDS replica1	2936	50	40	39278	49.5	Described in the text	60
GWALP 40 SDS replica1	4472	40	40	23775	39.9	Described in the text	61
GWALP 45 SDS replica1	5184	45	45	23810	44.5	Described in the text	62
GWALP 50 SDS replica1	4294	50	50	23860	49.5	Described in the text	63
GWALP 60 SDS replica1	3823	60	60	29237	59.0	Described in the text	64
GWALP 70 SDS replica1 – separate	3645	70	70	23523	69.4	Described in the text	65
GWALP 70 SDS replica2 – together	3000	70	70	23523	63.1	GWALP dimer 50 SDS replica1, 4294 ns	66
GWALP 70 SDS replica3 – together	3000	70	70	23523	69.6	GWALP 70 SDS replica2, 3000 ns	67
GWALP 70 SDS replica4 – together	3000	70	70	23523	69.6	GWALP 70 SDS replica3, 1500 ns	68
GWALP 70 SDS replica5 – separate	3000	70	70	23523	69.4	GWALP 70 SDS replica1, 1820 ns	69
GWALP 70 SDS replica6 – separate	3000	70	70	23523	69.8	GWALP 70 SDS replica1, 3640 ns	70
GWALP 70 SDS replica7 – together	3000	70	70	23523	69.4	GWALP 70 SDS replica3, 3000 ns	71
GWALP 70 SDS, bigger box, replica1 – separate	3000	70	70	39424	67.7	GWALP 70 SDS replica1, 3000 ns [d)]	

[a)] Average number of SDS molecules forming the micelle during simulation.
[b)] Origin of the starting configuration (name of the simulation, time of the frame).
[c)] All the simulation files including trajectories available from this reference.
[d)] Box dimensions were increased and additional water was added using gmx solvate

Supplementary Table 3. Simulated systems of SDS micelles without peptides

System	Length [ns]	SDS	Na^+	Water molecules	Saving frequency [ps]	Starting configuration[a]	Reference for the data[b]
Amber, TIP4P, 293 K	1678	60	60	16814	10	CHARMM, OPC, 362 ns	1
Amber, TIP4P, 293 K, high SF, replica1	50	60	60	16814	0.01	Amber, 500 ns	2
Amber, TIP4P, 293 K, high SF, replica2	50	60	60	16814	0.01	Amber, 1000 ns	
Amber, TIP4P, 293 K, high SF, replica3	50	60	60	16814	0.01	Amber, 1500 ns	
Amber, TIP4P, 307 K	1677	60	60	16814	10	CHARMM, OPC, 362 ns	3
Amber, TIP4P, 307 K, high SF, replica1	50	60	60	16814	0.01	Amber, 500 ns	4
Amber, TIP4P, 307 K, high SF, replica2	50	60	60	16814	0.01	Amber, 1000 ns	
Amber, TIP4P, 307 K, high SF, replica3	50	60	60	16814	0.01	Amber, 1500 ns	
CHARMM36, TIP3P, 293 K, replica0	214	60	60	16854	10	CHARMM-GUI	5
CHARMM36, TIP3P, 293 K, replica1	1809	60	60	16854	10	CHARMM-GUI	6
CHARMM36, TIP3P, 293 K, high SF, replica1	50	60	60	16854	0.01	CHARMM36, TIP3P, 500 ns	7
CHARMM36, TIP3P, 293 K, high SF, replica2	50	60	60	16854	0.01	CHARMM36, TIP3P, 1000 ns	
CHARMM36, TIP3P, 293 K, high SF, replica3	50	60	60	16854	0.01	CHARMM36, TIP3P, 1800 ns	
CHARMM36, TIP3P, 307 K, replica1	537	60	60	16854	10	CHARMM36, TIP3P, 293 K, replica1, 1800 ns	8
CHARMM36, TIP3P, 307 K, replica2	522	60	60	16854	10	CHARMM36, TIP3P, 307 K, replica1, 250 ns	9
CHARMM36, TIP3P, 307 K, replica3	546	60	60	16854	10	CHARMM36, TIP3P, 307 K, replica1, 500 ns	10
CHARMM36, TIP3P, 307 K, high SF, replica1	50	60	60	16854	0.01	CHARMM36, TIP3P, 250 ns	11
CHARMM36, TIP3P, 307 K, high SF, replica2	50	60	60	16854	0.01	CHARMM36, TIP3P, 500 ns	
CHARMM36, TIP3P, 307 K, high SF, replica3	50	60	60	16854	0.01	CHARMM36, TIP3P, 540 ns	
CHARMM36, OPC, 293 K, replica1	198	60	60	16393	10	CHARMM-GUI	12
CHARMM36, OPC, 293 K, replica2	362	60	60	16814	10	CHARMM-GUI	13
CHARMM36, OPC, 293 K, high SF, replica1	50	60	60	16814	0.01	CHARMM36, OPC, 100 ns	14
CHARMM36, OPC, 293 K, high SF, replica2	50	60	60	16814	0.01	CHARMM36, OPC, 200 ns	
CHARMM36, OPC, 293 K, high SF, replica3	50	60	60	16814	0.01	CHARMM36, OPC, 300 ns	
CHARMM36, OPC, 307 K, replica1	954	60	60	16814	10	CHARMM-GUI	15
CHARMM36, OPC, 307 K, high SF, replica1	50	60	60	16814	0.01	CHARMM36, OPC, 100 ns	16
CHARMM36, OPC, 307 K, high SF, replica2	50	60	60	16814	0.01	CHARMM36, OPC, 200 ns	
CHARMM36, OPC, 307 K, high SF, replica3	50	60	60	16814	0.01	CHARMM36, OPC, 300 ns	

[a)] Origin of the starting configuration (name of the simulation, time of the frame). [b)] All the simulation files including trajectories available from this reference.

Supplementary References

1. Nencini, R., DOI: 10.5281/zenodo.8182796 (2023). "Online; accessed 16 August 2023".
2. Nencini, R., DOI: 10.5281/zenodo.8182879 (2023). "Online; accessed 16 August 2023".
3. Nencini, R., DOI: 10.5281/zenodo.8182887 (2023). "Online; accessed 16 August 2023".

4. Nencini, R., DOI: 10.5281/zenodo.8182893 (2023). "Online; accessed 16 August 2023".
5. Nencini, R., DOI: 10.5281/zenodo.8182895 (2023). "Online; accessed 16 August 2023".
6. Regnier, M. Micelle size screening - eyqjd simulation - 50 sds - na neutralized - charmm36m - 310k - opc water model, DOI: 10.5281/zenodo.7695174 (2023). "Online; accessed 25 July 2023".
7. Nencini, R., DOI: 10.5281/zenodo.8183716 (2023). "Online; accessed 16 August 2023".
8. Nencini, R., DOI: 10.5281/zenodo.8183724 (2023). "Online; accessed 16 August 2023".
9. Nencini, R., DOI: 10.5281/zenodo.8183873 (2023). "Online; accessed 16 August 2023".
10. Nencini, R., DOI: 10.5281/zenodo.8183877 (2023). "Online; accessed 16 August 2023".
11. Nencini, R., DOI: 10.5281/zenodo.8183883 (2023). "Online; accessed 16 August 2023".
12. Regnier, M. Micelle size screening - hmff simulation - 40 sds - na neutralized - charmm36m - 310k - opc water model, DOI: 10.5281/zenodo.7696648 (2023). "Online; accessed 25 July 2023".
13. Nencini, R., DOI: 10.5281/zenodo.8183815 (2023). "Online; accessed 16 August 2023".
14. Nencini, R., DOI: 10.5281/zenodo.8183819 (2023). "Online; accessed 16 August 2023".
15. Regnier, M. Micelle size screening - hmff simulation - 45 sds - na neutralized - charmm36m - 310k - opc water model, DOI: 10.5281/zenodo.7728323 (2023). "Online; accessed 25 July 2023".
16. Nencini, R., DOI: 10.5281/zenodo.8183823 (2023). "Online; accessed 16 August 2023".
17. Nencini, R., DOI: 10.5281/zenodo.8183827 (2023). "Online; accessed 16 August 2023".
18. Regnier, M. Micelle size screening - hmff simulation - 50 sds - na neutralized - charmm36m - 310k - opc water model, DOI: 10.5281/zenodo.7728329 (2023). "Online; accessed 25 July 2023".
19. Nencini, R., DOI: 10.5281/zenodo.8183829 (2023). "Online; accessed 16 August 2023".
20. Nencini, R., DOI: 10.5281/zenodo.8183833 (2023). "Online; accessed 16 August 2023".
21. Nencini, R., DOI: 10.5281/zenodo.8252709 (2023). "Online; accessed 16 August 2023".
22. Nencini, R., DOI: 10.5281/zenodo.8252711 (2023). "Online; accessed 16 August 2023".
23. Nencini, R., DOI: 10.5281/zenodo.8252717 (2023). "Online; accessed 16 August 2023".
24. Nencini, R., DOI: 10.5281/zenodo.8252720 (2023). "Online; accessed 16 August 2023".
25. Nencini, R., DOI: 10.5281/zenodo.8252722 (2023). "Online; accessed 16 August 2023".
26. Nencini, R., DOI: 10.5281/zenodo.8252730 (2023). "Online; accessed 16 August 2023".
27. Regnier, M. Micelle size screening - hmff tail anchor simulation - 60 sds - na neutralized - charmm36m - 310k - opc water model, DOI: 10.5281/zenodo.7733867 (2023). "Online; accessed 25 July 2023".
28. Nencini, R., DOI: 10.5281/zenodo.8183835 (2023). "Online; accessed 16 August 2023".
29. Nencini, R., DOI: 10.5281/zenodo.8183841 (2023). "Online; accessed 16 August 2023".
30. Regnier, M. Micelle size screening - magaining 2 simulation - 50 sds - na neutralized - charmm36m - 310k - opc water model, DOI: 10.5281/zenodo.7695160 (2023). "Online; accessed 25 July 2023".
31. Nencini, R., DOI: 10.5281/zenodo.8183845 (2023). "Online; accessed 16 August 2023".
32. Nencini, R., DOI: 10.5281/zenodo.8183847 (2023). "Online; accessed 16 August 2023".
33. Nencini, R., DOI: 10.5281/zenodo.8183850 (2023). "Online; accessed 16 August 2023".
34. Nencini, R., DOI: 10.5281/zenodo.8183852 (2023). "Online; accessed 16 August 2023".
35. Nencini, R., DOI: 10.5281/zenodo.8183858 (2023). "Online; accessed 16 August 2023".
36. Regnier, M. Micelle size screening - gwalp tail anchor simulation - 40 sds - na neutralized - charmm36m - 310k - opc water model, DOI: 10.5281/zenodo.7736350 (2023). "Online; accessed 25 July 2023".
37. Regnier, M. Micelle size screening - gwalp tail anchor simulation - 45 sds - na neutralized - charmm36m - 310k - opc water model, DOI: 10.5281/zenodo.7736358 (2023). "Online; accessed 25 July 2023".
38. Regnier, M. Micelle size screening - gwalp tail anchor simulation - 50 sds - na neutralized - charmm36m - 310k - opc water model, DOI: 10.5281/zenodo.7736372 (2023). "Online; accessed 25 July 2023".

39. Nencini, R., DOI: 10.5281/zenodo.8183797 (2023). "Online; accessed 16 August 2023".

40. Nencini, R., DOI: 10.5281/zenodo.8183799 (2023). "Online; accessed 16 August 2023".

41. Nencini, R., DOI: 10.5281/zenodo.8183803 (2023). "Online; accessed 16 August 2023".

42. Nencini, R., DOI: 10.5281/zenodo.8183809 (2023). "Online; accessed 16 August 2023".

43. Regnier, M. Micelle size screening - eelab tail anchor dimer simulation - 50 sds - na neutralized - charmm36m - 310k - opc water model, DOI: 10.5281/zenodo.7784246 (2023). "Online; accessed 25 July 2023".

44. Regnier, M. Micelle size screening - yfis1 tail anchor dimer simulation - 50sds - na neutralized - charmm36m - 310k - opc water model, DOI: 10.5281/zenodo.7782672 (2023). "Online; accessed 25 July 2023".

45. Regnier, M. Micelle size screening - gwalp tail anchor dimer simulation - 40 sds - na neutralized - charmm36m - 310k - opc water model, DOI: 10.5281/zenodo.7774646 (2023). "Online; accessed 25 July 2023".

46. Regnier, M. Micelle size screening - gwalp tail anchor dimer simulation - 45 sds - na neutralized - charmm36m - 310k - opc water model, DOI: 10.5281/zenodo.7773903 (2023). "Online; accessed 25 July 2023".

47. Regnier, M. Micelle size screening - gwalp tail anchor dimer simulation - 50 sds - na neutralized - charmm36m - 310k - opc water model, DOI: 10.5281/zenodo.7773896 (2023). "Online; accessed 25 July 2023".

48. Regnier, M. Micelle size screening - gwalp tail anchor dimer simulation - 60 sds - na neutralized - charmm36m - 310k - opc water model, DOI: 10.5281/zenodo.7774648 (2023). "Online; accessed 25 July 2023".

49. Regnier, M. Micelle size screening - gwalp tail anchor dimer simulation - 70 sds - na neutralized - charmm36m - 310k - opc water model, DOI: 10.5281/zenodo.7782287 (2023). "Online; accessed 25 July 2023".

50. Nencini, R., DOI: 10.5281/zenodo.8183728 (2023). "Online; accessed 16 August 2023".

51. Nencini, R., DOI: 10.5281/zenodo.8183753 (2023). "Online; accessed 16 August 2023".

52. Nencini, R., DOI: 10.5281/zenodo.8183760 (2023). "Online; accessed 16 August 2023".

53. Nencini, R., DOI: 10.5281/zenodo.8183789 (2023). "Online; accessed 16 August 2023".

54. Nencini, R., DOI: 10.5281/zenodo.8183791 (2023). "Online; accessed 16 August 2023".

55. Nencini, R., DOI: 10.5281/zenodo.8183795 (2023). "Online; accessed 16 August 2023".

56. Nencini, R., DOI: 10.5281/zenodo.8254318 (2023). "Online; accessed 17 August 2023".

57. Nencini, R., DOI: 10.5281/zenodo.8254382 (2023). "Online; accessed 17 August 2023".

58. Nencini, R., DOI: 10.5281/zenodo.8254393 (2023). "Online; accessed 17 August 2023".

59. Nencini, R., DOI: 10.5281/zenodo.8254420 (2023). "Online; accessed 17 August 2023".

60. Regnier, M. Empty micelle - 60 sds - na neutralized - charmm36m - 293k - tip3p water model, DOI: 10.5281/zenodo.7819608 (2023). "Online; accessed 25 July 2023".

61. Nencini, R., DOI: 10.5281/zenodo.8254435 (2023). "Online; accessed 17 August 2023".

62. Nencini, R., DOI: 10.5281/zenodo.8254441 (2023). "Online; accessed 17 August 2023".

63. Nencini, R., DOI: 10.5281/zenodo.8254450 (2023). "Online; accessed 17 August 2023".

64. Nencini, R., DOI: 10.5281/zenodo.8254457 (2023). "Online; accessed 17 August 2023".

65. Nencini, R., DOI: 10.5281/zenodo.8254459 (2023). "Online; accessed 17 August 2023".

66. Nencini, R., DOI: 10.5281/zenodo.8254479 (2023). "Online; accessed 17 August 2023".

67. Regnier, M. Empty micelle - 60 sds - na neutralized - charmm36m - 293k - opc water model - replica 0, DOI: 10.5281/zenodo.7788642 (2023). "Online; accessed 25 July 2023".

68. Regnier, M. Empty micelle - 60 sds - na neutralized - charmm36m - 293k - opc water model - replica 1, DOI: 10.5281/zenodo.7788679 (2023). "Online; accessed 25 July 2023".

69. Nencini, R., DOI: 10.5281/zenodo.8254495 (2023). "Online; accessed 17 August 2023".

70. Nencini, R., DOI: 10.5281/zenodo.8254514 (2023). "Online; accessed 17 August 2023".

71. Nencini, R., DOI: 10.5281/zenodo.8254520 (2023). "Online; accessed 17 August 2023".
